# Value of species and the evolution of conservation ethics

**DOI:** 10.1098/rsos.181038

**Published:** 2018-11-21

**Authors:** Darragh Hare, Bernd Blossey, H. Kern Reeve

**Affiliations:** 1 Department of Natural Resources, Cornell University, Fernow Hall, Ithaca, NY 14853, USA; 2 Department of Neurobiology and Behavior, Cornell University, Mudd Hall, Ithaca, NY 14853, USA

**Keywords:** altruism, biodiversity conservation, conservation ethics, cooperation, evolution of morality, Hamilton's rule

## Abstract

The theory of evolution by natural selection can help explain why people care about other species. Building upon recent insights that morality evolves to secure fitness advantages of cooperation, we propose that conservation ethics (moral beliefs, attitudes, intuitions and norms regarding other species) could be adaptations that support cooperation between humans and non-humans. We present eco-evolutionary cost–benefit models of conservation behaviours as interspecific cooperation (altruism towards members of other species). We find that an evolutionary rule identical in structure to Hamilton's rule (which explains altruistic behaviour towards related conspecifics) can explain altruistic behaviour towards members of other species. Natural selection will favour traits for selectively altering the success of members of other species (e.g. conserving them) in ways that maximize inclusive fitness return benefits. Conservation behaviours and the ethics that evolve to reinforce them will be sensitive to local ecological and socio-cultural conditions, so will assume different contours in different places. Difficulties accurately assessing costs and benefits provided by other species, time required to adapt to ecological and socio-cultural change and barriers to collective action could explain the apparent contradiction between the widespread existence of conservation ethics and patterns of biodiversity decline globally.

## 1. Background

Why should we care about biodiversity loss and ecological change? Which species should we conserve, and why? Are species valuable simply because they contribute to human well-being, or also for their own sake? To what extent should people consider the interests of non-humans in conservation? How we answer these enduring moral questions will influence our impacts on ecosystems that support human and non-human life [1]. Our answers will depend on how we understand the causal origins and scope of human morality, and how we interpret relationships between humans and other species.

Claims that humans have profound moral concern for other species might ring hollow as human activities continue to drive biodiversity declines globally [2–4]. Nevertheless, conservation ethics (individual-level moral beliefs, attitudes and intuitions, as well as population-level social norms regarding other species) appear to be widespread. Concerns about unsustainable human impacts on ecosystems [3,5–8] and calls for a concomitant recalibration of values and institutions [9–13] suggest that the moral dimensions of ecological change and biodiversity loss resonate deeply. Moral commitments to protect, conserve and respect other species are evident in social norms and cultural traditions around the world and over time [14–17], and in contemporary individual moral beliefs, attitudes and intuitions across societies [18–21]. However, fundamental disagreements about why conservation is important and the nature of human obligations to other species [22–24] cast doubt on whether conservation ethics will be able to inspire actions that successfully reduce or reverse biodiversity loss globally.

Members of the public in several countries believe that non-human species have both instrumental value (derived from benefits they provide to humans) and intrinsic or non-instrumental value (over and above benefits they provide to humans) [25], but assign them different relative importance [18–21,26–28]. Even conservation professionals, united by a foundational commitment to the value of biodiversity, are divided over which species are valuable, what type of value they have, and to whom they are valuable [24,29–32]. Despite recognition that simple categories such as instrumental and intrinsic value cannot capture the full diversity of conservation ethics [24,33], and calls for unity between opposing moral justifications for conservation [29,34–36], philosophical differences persist and impede conservation efforts [31].

Thoughtful reflection and debate about moral justifications for biodiversity conservation, informed by empirical research, can provide crucial guidance on balancing human interests with interests of other species. However, conservation ethics at the level of both individual attitudes and social norms have deep and complex psychological and cultural roots, so can be stubbornly resistant to change [37,38]. Continuing to rehearse arguments about ‘correct’ moral justifications for conservation is likely to deepen entrenched positions rather than inspire solutions. Because human behaviour reflects evolved solutions to adaptive challenges that arise in particular social and ecological environments [14,39–42], a fresh approach that integrates ecology and evolution into our understanding of human morality could provide novel insights into the origins of and justifications for conservation ethics.

Research across disciplines indicates that morality (beliefs, attitudes, intuitions and norms about what is right and wrong) is a set of adaptations favoured by natural selection to regulate behaviour in ways that promote mutually beneficial cooperation [43–46] in recurrent non-zero-sum interactions (i.e. when it is possible to produce ‘win–win’ outcomes). In such interactions, cooperators receive more favourable fitness outcomes than non-cooperators [47]. Cooperation is therefore favoured over non-cooperation within groups of frequently interacting individuals, and groups of cooperators outcompete groups of non-cooperators, so traits promoting adherence to cooperative rules are more likely to persist and spread [48]. Cooperation is so evolutionarily powerful that it is a fundamental component of the behavioural repertoires of group-living species [49] and explains much of how human societies are structured [50]. Moral beliefs, attitudes and intuitions nudge individuals towards cooperative behaviours, bolstering fitness advantages of cooperation [45,51].

While so far the evolutionary study of morality has focused on moral behaviour towards members of the same species, its basis in cooperation suggests that it could also explain moral behaviour towards members of other species. Interspecific cooperation is widespread [52,53], and humans frequently cooperate with other species, from gut microbiota that keep us healthy, to animals that help us make a living, to plants we cultivate and animals whose habitats we maintain. There is therefore no *a priori* reason why morality could not evolve to promote fitness advantages of interspecific cooperation.

To investigate this possibility, we develop general evolutionary cost–benefit models of interspecific cooperation that can apply to human conservation behaviour. Specifically, we ask whether altruism (improving another's fitness at some initial fitness cost to oneself [54]), a form of cooperation observed in many taxa and a defining feature of human morality [43,55], can also be adaptive (i.e. ultimately increase inclusive fitness) when directed towards members of other species, even if they have not been naturally selected to conditionally repay the altruistic act. Just as many aspects of morality have evolved to promote cooperation in recurrent social interactions within groups of humans, so might conservation ethics have evolved to promote cooperation in recurrent ecological interactions between humans and members of other species. A foundational account of how and why conservation behaviours evolve could help explain why conservation ethics exist, and why they vary and shed light on the apparent contradiction between the widespread occurrence of conservation ethics and patterns of biodiversity decline globally.

## 2. Models

### 2.1. Conservation behaviours as interspecific altruism

Conservation behaviours involve an individual of one species paying some cost to take an action that will benefit at least one individual of another species [42,56], for example, by restraining consumption, modifying environments or directly providing resources. Conservation behaviours can therefore be considered altruistic because one individual pays an initial personal fitness cost to provide a benefit to one or more others [54]. Like any other behaviour, conservation behaviours will be favoured by natural selection if they ultimately increase the inclusive fitness of individuals who engage in them and will spread in a population if they increase the inclusive fitness of individuals who engage in them relative to individuals who do not [54]. Because inclusive fitness takes into account the fitness of an altruist's relatives as well as the altruist's personal fitness, an altruist need not receive any personal fitness gains for altruism to evolve and spread. Behaviours can spread genetically, culturally or through gene–culture interactions [57], so long as transmission rules have evolved to maximize inclusive fitness. Interactions described by our models do not necessarily involve humans (electronic supplementary material, appendix A), so we make no assumptions about the species to which interactants belong.

We model conservation behaviours as actions taken by individuals. In reality groups of individuals can exhibit coordinated conservation behaviours, but because selection will work on differential inclusive fitness outcomes among individuals, we restrict our models accordingly. In the discussion, we explore the implications of this restriction. In order to capture a broad range of realistic conservation behaviours, we allow recipients of conservation behaviours to be individuals or groups such as populations.

We begin by asking whether it could ever be adaptive for a focal individual to take some action that will alter the success, *a*, of a recipient of a different species by an amount *x*. (Table 1 contains detailed definitions of all variables in our models.) The action in question will carry some initial personal fitness cost, *c*, to the focal individual. Altering the recipient's success will affect the focal individual's inclusive fitness, *w*, scaled by the ‘ecological relatedness', *r*, between the focal individual and the recipient. Ecological relatedness is a multiplier that converts a change in the recipient's success into a change in the focal individual's inclusive fitness. Increasing a recipient's success will generate a positive return effect on the focal individual's inclusive fitness when *r* is positive, and a negative return effect when *r* is negative. For example, crops could have a positive *r* value, because increasing their success is likely to provide positive inclusive fitness returns, whereas poisonous plants, pathogens or dangerous animals could have a negative *r* value because increasing their success might provide negative inclusive fitness returns by amplifying the risks they present. The absolute value for *r* represents the strength of positive or negative return effects on the focal individual's inclusive fitness.

**Table 1.** Definitions, possible conditions and explanations for all model variables, grouped by model and listed in the order they appear in the text.

model	variable	definition	possible conditions	explanation
1. conservation behaviours as interspecific altruism	*a*	baseline success of recipient	always positive	recipient will always have some baseline level of success
	*x*	amount by which focal individual changes recipient's success from its baseline success, *a*	positive	increases recipient's total success above baseline success (altruism, conservation)
			negative	decreases recipient's total success below baseline success (spite, persecution)
			neutral (zero)	does not change recipient's total success from baseline success
	*c*	fitness cost of *doing something*	always positive	*doing something* will always carry some fitness cost to focal individual
	*w*	inclusive fitness of focal individual	always positive	focal individual will always have some inclusive fitness
	*r*	ecological relatedness between focal individual and recipient, i.e. a multiplier that converts *x* (change in recipient's success, *a*) into change in focal individual's inclusive fitness, *w*. This can be expressed in two equivalent ways: (i) average effect on the focal individual's inclusive fitness, *w*, of altering recipient's success by an amount *x*, and (ii) slope of the expected return on focal individual's inclusive fitness, *w*, as a function of the change of amount *x* in recipient's success	positive	increasing recipient's success generates a positive return effect on focal individual's inclusive fitness; decreasing recipient's success generates a negative return effect on focal individual's inclusive fitness
			negative	increasing recipient's success generates a negative return effect on focal individual's inclusive fitness; decreasing recipient's success generates a positive return effect on focal individual's inclusive fitness
			neutral (zero)	increasing or decreasing recipient's success generates no return effect on focal individual's inclusive fitness
	*z*	focal individual's baseline inclusive fitness, regardless of whether it *does something* or *does nothing*	always positive	focal individual will always have some baseline inclusive fitness
2. indirect effects of downstream ecological interactions	*r*′	net ecological relatedness between focal individual and recipient, incorporating all downstream ecological effects	positive	increasing recipient's success generates a positive return effect on focal individual's inclusive fitness; decreasing recipient's success generates a negative return effect on focal individual's inclusive fitness
			negative	increasing recipient's success generates a negative return effect on focal individual's inclusive fitness; decreasing recipient's success generates a positive return effect on focal individual's inclusive fitness
			neutral (zero)	increasing or decreasing recipient's success generates no return effect on focal individual's inclusive fitness
	*u*	proportion of initial investment that alters success of downstream recipients; quantifies strength and sign of indirect effect on downstream recipient of investment in initial recipient	positive	when effect on initial recipient (*x*) is positive, indirect effect on downstream recipient is also positive; when effect on initial recipient is negative, effect on downstream recipient is also negative
			negative	when effect on initial recipient (*x*) is positive, effect on downstream recipient is negative; when effect on initial recipient is negative, effect on downstream recipient is positive
3. continuous effort and nonlinear returns	*y*	level of effort invested in altering recipient's success	always positive	higher value for *y* means that the focal individual invests more effort in altering recipient's success
	*d*	determines whether investment increases or decreases recipient's success	positive	increased investment increases recipient's success
			negative	increased investment decreases recipient's success
	*v*	determines how sensitively an increasing investment affects the recipient's success	always positive	higher value for *v* amplifies effects of investment on recipient's success (minimal non-zero *y* scaled to be 1 or greater)
	*c*	determines cost rate of investment *y*	positive	higher values for *c* indicate a higher cost for a given investment, *y*
	*t*	determines how sensitively an increasing investment affects the focal individual's cost	always positive	higher value for *t* increases the cost of investment (minimal non-zero *y* scaled to be 1 or greater)
	y*	optimal level of effort in terms of focal individual's inclusive fitness	always non-negative	by investing y*, focal individual maximizes its net inclusive fitness; corresponds with dashed lines in figure 1

It will be adaptive for the focal individual to take an action that alters the recipient's success when

2.1 z+r(a+x)−c > z+ra,

where *z* is the focal individual's baseline inclusive fitness, regardless of whether it *does something* or *does nothing*. The left-hand side of this inequality represents the focal individual's inclusive fitness, *w*, for *doing something* (*w* = *z* + *r*(*a* + *x*) − *c*) and the right-hand side represents the focal individual's inclusive fitness for *doing nothing*, i.e. not taking the action in question (*w* = *z* + *ra*).

The inequality simplifies to

2.2 rx>c.

Therefore, it will be adaptive for a focal individual to take an action that affects a recipient's success (*do something*) when ecological relatedness between the recipient and the focal individual, multiplied by the strength of effect on the recipient's success, exceeds the cost of taking the action under consideration. We call this the adaptive conservation rule (ACR). We do not assume that the focal individual consciously calculates costs and benefits, only that selection will favour traits that promote *doing something* when ACR is satisfied and *doing nothing* when ACR is not satisfied.

### 2.2. Indirect effects of downstream ecological interactions

Actions that positively or negatively affect the success of members of one species are likely also to have positive or negative indirect effects on the success of members of additional species ecologically connected to the first [58–60]. These indirect effects will also impact the focal individual's inclusive fitness, so will determine whether *doing something* will be adaptive.

Suppose a focal individual alters the success of an initial recipient by an amount *x*, which in turn affects the success of *n* other ‘downstream’ recipients belonging to additional species. The *i*th such downstream recipient experiences an indirect effect of the focal individual's action *u_i_x*, that positively or negatively changes the downstream recipient's success from its baseline success, *a_i_*, to *a_i_* + *u_i_x*, where *u* quantifies the strength and sign of indirect effects. Each such downstream recipient provides an inclusive fitness return *r_i_*(*a_i_* + *u_i_x*) to the focal individual. The initial recipient, indexed as zero, provides a return *r*_0_(*a*_0_ + *u*_0_*x*) to the focal individual. We conservatively assume that all such returns additively combine. Returns might multiplicatively combine, which would amplify the effects of downstream interactions. Under this additive assumption, the total (net) fitness return to the focal individual is

2.3 $$\overset{n}{\underset{i=0}{\sum }}r_{i}\;*\;(a_{i}+u_{i}x),$$

where *u*_0_ = 1*.*

Since taking the action with effect *x* necessarily entails a cost *c* to the focal individual, then the action will be favoured if

2.4 $$\overset{n}{\underset{i=0}{\sum }}r_{i}\;*\;(a_{i}+u_{i}x)-\overset{n}{\underset{i=0}{\sum }}r_{i}a_{i}>c,$$

which simplifies to

2.5 $$\overset{n}{\underset{i=0}{\sum }}r_{i}u_{i}x>c.$$

This is equivalent to ACR if we define the initial recipient's net ecological relatedness *r′* to the focal individual as

2.6 $$r^{\prime }=\overset{n}{\underset{i=0}{\sum }}r_{i}u_{i},$$

entailing that altering the success of the initial recipient species by *x* is favoured if *r*′*x* > *c*, just as in the simple formulation of ACR in model 1. This reformulation of ACR is more ecologically realistic because it accommodates positive and negative indirect effects on all downstream recipients (i.e. broader cascading effects on the ecological community), which will mediate the net inclusive fitness consequences of *doing something*.

### 2.3. Continuous effort and nonlinear returns

Models 1 and 2 treat conservation behaviours as discrete acts of investment in members of other species (*doing something*). However, it would be more realistic to model conservation behaviours as investments of continuously varying effort, *y*, in altering a recipient's success, and to allow costs of and returns on such investments to be nonlinear continuous functions.

For example, increasing effort might affect the recipient's success nonlinearly, according to the function *dy^v^*, where *d* and *v* are constants, *v* > 0. By replacing *x* in model 2 with this new function, *dy^v^*, the return inclusive fitness benefit to the focal individual for *doing something to an extent y* would be *r′*(*a* + *dy^v^*). Negative values for *d* represent investments that decrease the recipient's success, and positive values for *d* represent investments that increase the recipient's success. If 0 < *v* < 1, then increasing investment will have diminishing marginal effects on the recipient's success, and if *v* > 1, increasing investment will have accelerating marginal effects on the recipient's success. Increasing investment might also affect the cost to the focal individual nonlinearly, according to the function *cy^t^*, where *c* and *t* are positive constants. If 0 < *t* < 1, then increasing investment will yield diminishing increases in costs, and if *t* > 1, increasing investment will yield accelerating costs.

By further modifying model 2 to also account for nonlinear costs of investment, the focal individual's net inclusive fitness becomes

2.7 $$w=z+r^{\prime }(a+dy^{v})-cy^{t}.$$

This retains the basic structure of models 1 and 2 but is more realistic because it incorporates nonlinear costs as well as nonlinear inclusive fitness returns of investments.

**Figure 1.**
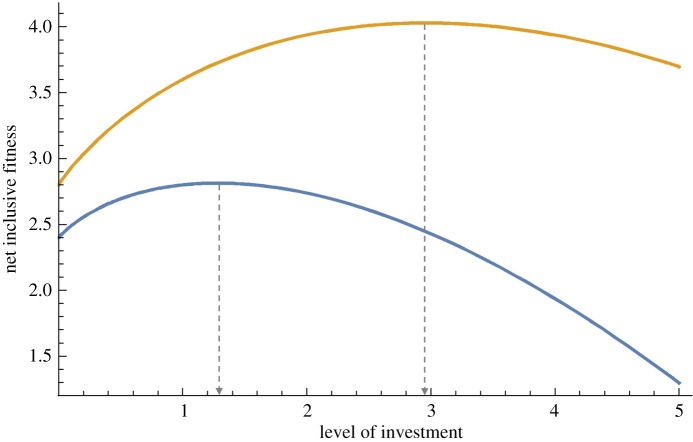
Net inclusive fitness returns for continuous investment of effort under different conditions of net ecological relatedness. Nonlinear net inclusive fitness, *w*, returns for the level of effort, *y*, such that $w=z+r^{\prime }(a+dy^{v})-cy^{t}.$ For the blue curve, values are set to *r*′ = 1.4, *a* = 1, *c* = 1, *d* = 1, *t* = 1.3, *v* = 1. For the orange curve, all values are the same except *r*′ = 1.8. Higher *r*′ (orange curve) yields greater net inclusive fitness returns for a given level of investment. Dashed vertical lines indicate the optimal level of investment y*, which increases as net ecological relatedness, *r*′, increases. Focal individuals can maximize inclusive fitness returns by focusing their investments in members of species with higher *r*′.

If costs rise faster than benefits of investment as investment increases (*t* > *v*), the focal individual's net inclusive fitness will reach a peak at an intermediate optimal level of investment (figure 1). Natural selection will favour investing at this optimal level, y*, which is given by

2.8 $$y^{*}=e^{\ln[r^{\prime }dv/(tc)]/(t-v)}.$$

The optimal level of investment increases as the product of benefit-related parameters *r′dv* increases and as cost-related parameters *c* and *t* decrease.

## 3. Results

Any action that alters the success of members of another species will also generate return effects on the inclusive fitness of the individual taking that action (unless net ecological relatedness *r′* is exactly zero). Natural selection will operate on different strategies for altering the success of members of other species, favouring those strategies that produce the greatest net inclusive outcomes. Net inclusive fitness outcomes will reflect the cost of taking an action as well as the sum of all direct and indirect return effects. Additive indirect ecological effects (incorporated into *r′*) could increase or decrease the magnitude of direct effects (represented by *r*), in some cases strongly enough to reverse the sign from positive to negative or negative to positive.

**Figure 2.**
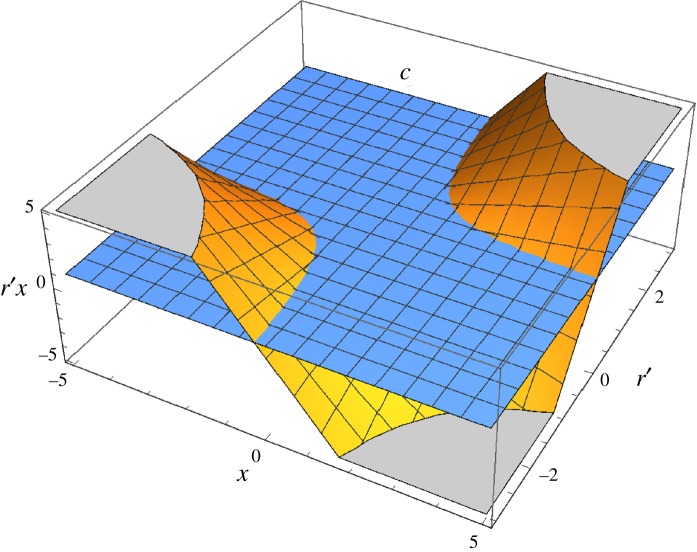
Adaptive strategies under the adaptive conservation rule (ACR). Regions in which *doing something* is favoured because *r*′*x* (net ecological relatedness multiplied by the effect of a given action on the recipient's success) exceeds cost, *c*. Orange surfaces are *r*′*x* and blue plane is *c*. *Doing something* is favoured in both scenarios in which orange surfaces lie above the blue plane. In the first scenario (upper right quadrant), it will be adaptive for the focal individual to increase the recipient's success (i.e. engage in conservation behaviour). In the second scenario (lower left quadrant), it will be adaptive for the focal individual to decrease the recipient's success (i.e. engage in persecution behaviour). For simplicity, this figure uses notation from model 2.

ACR represents a general rule for maximizing net inclusive fitness outcomes of altering the success of members of other species. Adaptive strategies under ACR (figure 2) include paying a cost to positively affect a recipient's success (altruism, positive value for *x*, conservation behaviours) as well as paying a cost to negatively affect a recipient's success (spite, negative value for *x*, persecution behaviours) [61]. Similarly, in model 3, optimal levels of investment increase the greater the degree that investment increases the success of beneficial species (positive values for both *r′* and *d*) or depresses the success of harmful species (negative values for both *r′* and *d*). Optimal strategies will be sensitive to nonlinear payoff functions (figure 1) to prevent over-investment in a recipient, i.e. affecting a recipient's success so much that doing so produces a net negative inclusive fitness outcome for the focal individual.

ACR is homomorphic to Hamilton's rule [54], which explains why individuals will act altruistically towards conspecifics. Hamilton's rule states that it will be adaptive for a focal individual to pay a cost to provide some benefit to a recipient, so long as benefits to the recipient multiplied by genetic relatedness between the two is greater than the cost to the focal individual (*rb* > *c*). When Hamilton's rule is satisfied, altruistic individuals pay a personal fitness cost but receive a net inclusive fitness gain. The same is true when ACR is satisfied: expected inclusive fitness consequences of *doing something* exceed expected inclusive fitness consequences of *doing nothing*, so *doing something* is favoured even though it involves an initial personal fitness cost. However, in our interspecific models, the factor that scales effects of investments to the focal individual's inclusive fitness is ecological relatedness, not genetic relatedness. Genetic relatedness is a fixed probability that individuals share alleles, but ecological relatedness and net ecological relatedness can each take on different values in different contexts.

Models 1 and 2 provide a general theory of the inclusive fitness value of genetically unrelated organisms, and model 3 explains how focal individuals can maximize their inclusive fitness returns by selectively affecting the success of members of other species. Under all three formulations of ACR, focal individuals can maximize their inclusive fitness by working harder to alter the success of members of species with larger absolute values of ecological relatedness (*r* or *r′*). This includes increasing members of other species' success when net ecological relatedness (*r′*) is positive and decreasing their success when net ecological relatedness is negative.

## 4. Discussion

By describing how natural selection can favour interspecific cooperation in the form of ACR, we demonstrate one way in which selective conservation behaviours can be adaptive. Our models therefore reveal a possible evolutionary basis for conservation ethics: adaptive conservation behaviours will spread in a population and conservation ethics will evolve to bolster resultant fitness advantages. Like morality more generally [43,45], conservation ethics could be rooted in cooperative behaviour.

### 4.1. Interspecific cooperation

By showing that *doing something* can be adaptive when values for *r′* and *x* are low, when effects are indirect, and when no specific return behaviour is required, our models extend the logic of Hamilton's rule to a broader set of biological conditions. When ACR is satisfied, return inclusive fitness benefits, scaled by net ecological relatedness, exceed initial personal fitness costs of altruistic behaviours. Such return benefits unite ACR mathematically with two established evolutionary accounts of altruism, although they differ in precisely how the actor obtains return benefits: reciprocal altruism [55], in which return benefits result from repeated interactions among conditionally reciprocating altruists; and kin-selected altruism [54], in which return benefits accrue to altruists through relatives sharing the gene for altruism. Without return benefits, altruistic behaviours would incur costs but provide no inclusive fitness gains, so would not be favoured by selection.

We do not intend our models to replace existing explanations of mutualism, in which species co-evolve specific behaviours to provide benefits to each other [62]. Previous studies have described the mathematics of mutualism [62–65], some of which have also drawn parallels to Hamilton's rule [66,67]. Because our models do not assume that the focal individual and recipient belong to species that interact frequently or have been under selection to provide reciprocal benefits, they apply more broadly than just to mutualisms. Our models can accommodate mutualisms, which would emerge through strong positive selection for higher values for *r*. Our models are distinctive because they can (i) extend to antagonistic behaviours towards members of other species, represented by negative values for *x* and *d*, (ii) take into account indirect effects on an indefinite number of downstream species and ecological interactions, so extend beyond interactions between a focal individual and recipients of a single different species and (iii) apply to an adaptive form of unilateral interspecific altruism, i.e. cases in which recipients have not necessarily evolved specific behaviours to provide return benefits (sometimes referred to as by-product mutualism [62]). Even without such specific behaviours, in our models inclusive fitness benefits can accrue to the focal individual by virtue of broader direct and indirect ecological consequences of investing in a recipient's success within a larger ecological community. Inclusive fitness gains could offset the cost of an initial investment when return benefits accrue to the focal individual (short-term reduction but lifetime increase in the focal individual's personal fitness) or to the focal individual's relatives (lifetime reduction in focal individual's personal fitness but net inclusive fitness gain through increased fitness of relatives).

### 4.2. Ecology, local adaptation and cultural diversity

Although our models could apply to cooperation between interactants of potentially any species, we are especially interested in whether they can explain observed patterns in human conservation behaviours and ethics. ACR sheds light on some factors that may have influenced our ancestors' conservation behaviours and continue to influence our own. Acting in accordance with ACR would help humans maximize inclusive fitness benefits of direct and indirect interactions with the species they encounter, so conservation behaviours will over time become adapted to local ecologies [68–70]. We propose that conservation ethics are also part of this dynamic system, regulating individual and collective behaviours in ways that promote and reinforce adaptive strategies for interacting with members of non-human species.

From this perspective, conservation ethics are not intellectual luxuries divorced from or imposed upon ecology, but components of evolved survival strategies sensitive to ecological and socio-cultural conditions. We emphasize that local adaptation does not necessarily imply evolved genetic differences among human populations with different conservation ethics, because local adaptation could reflect a general adaptive rule for (consciously or unconsciously) flexibly assessing costs and benefits of different behaviours in different socio-cultural and ecological contexts. Individuals could acquire particular expressions of that rule through trial and error or observing others. Innate or culturally acquired propensities to favour certain species over others encoded in beliefs, attitudes and intuitions could promote adaptive conservation behaviours at the level of the individual. Social norms, formal and informal rules that regulate behaviour within groups, could reflect long-run costs and benefits of acting altruistically towards members of other species and reinforce adaptive individual conservation behaviours at the population level.

We do not suggest that conservation behaviours and ethics will always and everywhere be perfectly fine-tuned to local conditions. Difficulties accurately assessing costs and benefits other species provide, time required to adapt to ecological and socio-cultural change, and barriers to collective action might cause conservation ethics to be suboptimal. These factors could help explain the apparent contradiction between the widespread existence of conservation ethics and patterns of biodiversity decline globally.

A key prediction of our models is that conservation ethics will be geared towards conserving locally important species (those with a high positive value for *r* or *r′*). People conserve species of particular local importance to human well-being by assigning them special cultural status, for example, corn (*Zea mays*) among the Q'eqchí and Poqomchí peoples of Alta Verapaz in Guatemala [71], and fig trees (*Ficus* spp.) in several societies in Africa and Asia [40]. By repeatedly selecting for traits that provide greatest return benefits, for example, through dispersal, cultivation, intensification or domestication [72–75], people could consciously or unconsciously increase *r* or *r′* values of species over time.

Local adaptation implies that different human populations could assign a given species different moral status depending on its local *r′* value, which will reflect social and cultural practices as well as ecological community composition. Differential *r′* values could potentially explain why moral attitudes towards other species vary between and within human societies [18,76,77]. For example, in India people who live in close proximity to species that can negatively impact their well-being, such as tigers (*Panthera tigris*) and king cobras (*Ophiophagus hannah*), appreciate those species less than people who live further from them [78].

Our models also predict that under some conditions selection will favour anti-conservation behaviours (i.e. decreasing a recipient's success through persecution or extirpation, figure 2). Model 3 predicts that people will invest more effort in conserving or persecuting species when doing so will provide greater net inclusive fitness returns (right-shifted peaks on net inclusive fitness return curves, figure 1) because of larger absolute magnitudes of ecological relatedness. We would therefore expect persecution ethics to evolve and perhaps find expression in people's support for efforts to limit the deleterious effects of invasive or other species that disrupt established ecological dynamics [79,80]. In extreme cases, acting in accordance with ACR could lead people to extirpate categorically harmful species (negative values for *r* and *r′*), such as the bacterial agent of anthrax (*Bacillus anthracis*).

Political tensions regarding presence and abundance of controversial species such as large carnivores could reflect their differential *r* and *r′* values to different people. Although large carnivores can be extremely politically divisive, people tend to express favourable moral attitudes towards them [27,76,81] and invest large amounts of money and effort in conserving them despite significant habitat requirements and difficulty of effective conservation interventions [82,83]. Large investments will be worthwhile from an evolutionary perspective when the net inclusive fitness benefits are sufficiently high. For example, this could apply to conserving or restoring large carnivores because they provide public health benefits [60] or increase community diversity by exerting top-down structuring effects that regulate consumer species at lower trophic levels [84]. People develop practices and technologies including physical deterrents or monitoring systems that mitigate the harmful effects of large carnivores such as intense predation on domestic animals or other beneficial species [85,86]. These practices and technologies effectively diminish large carnivores' negative *r* values and thereby increase their *r′* values.

If conservation ethics are locally adapted, we would expect justifications for why certain behaviours towards members of other species are right or wrong, required or forbidden also to vary between human societies. Such justifications form part of societies’ larger worldviews, including their cosmology, their interpretation of the place of humans in nature, and their more general moral systems [11,87], for example, different religious expressions of moral responsibilities towards non-humans [88]. Justifications for conservation can be scrutinized in terms of ethical assumptions and internal consistency, whether they emphasize economic value [89], instrumental value [90], intrinsic value [91] or avoidance of supernatural punishment [41]. From an evolutionary standpoint, justifications that effectively promote adaptive conservation behaviours can persist regardless of whether they are able to withstand rigorous philosophical or logical scrutiny.

Over time, adaptive conservation behaviours become supported by rules of thumb, heuristics that condense complex local ecological knowledge into clear and simple guidance [40], and social taboos, informal prohibitions of particular behaviours [92]. Across societies, rules of thumb and social taboos permanently or seasonally restrict access to locally important species and protect places associated with high biodiversity [14,92–97]. Such conservation-oriented rules of thumb and taboos safeguard benefits from local ecosystems but do not always originate from deliberate attempts to conserve species or communities [92]. In this respect, they are functionally similar to rules promoting other adaptive behaviours, such as incest avoidance and food taboos, which have evolved across human societies to minimize risks associated with inbreeding and food-borne illness [98,99]. Justifications for such rules regulate behaviour to maximize fitness in particular socio-cultural and ecological contexts, even though they often include no mention of adaptive advantages and are not always fully consciously understood by people who adhere to them [100].

### 4.3. Obvious and non-obvious benefits

Benefits other species and biodiversity more generally provide to humans can be subtle and diffuse [6,60,101]. As long as feedbacks between an individual's behaviour, ecological consequences and inclusive fitness are sufficiently strong, natural selection will favour conserving species with a sufficiently positive *r′* value, even when those species' apparent direct impact on humans is neutral or negative. Errors in ascertaining benefits provided by some species (overlooking non-obvious benefits, and therefore miscalculating *r′*) could lead to actions that are detrimental to human well-being. Historical examples of such errors include extirpations of apex consumers that initiated trophic cascades resulting in significant changes to herbivore and autotroph populations [58], and attempts to eradicate scavengers or microbial communities that were beneficial to human health [60,102]. In these cases, people negatively affected the success of species with positive *r′* values, an action that could not satisfy ACR (figure 2).

Because *r* represents direct benefits, we would expect natural selection to favour moral beliefs, attitudes and intuitions promoting conservation of species with obviously high *r* values, such as those based on instrumental value to humans. To prevent mistakes of focusing exclusively on *r*, especially when *r′*, which incorporates indirect ecological benefits, is vastly more positive, we would also expect natural selection to favour beliefs, attitudes and intuitions promoting conservation of species whose benefits to humans are not obvious or even completely opaque. Assigning such species intrinsic or non-instrumental value could serve this purpose. People across societies do assign an intrinsic or non-instrumental value to species, ecosystems or ‘nature’ more generally, although specific articulations vary [20,26,27,103]. Moral maxims urging respect for members of non-human species for reasons other than their immediately obvious instrumental value have emerged in several traditions and schools of thought. They are expressed in terms appropriate to the society from which they emerge, for example, mechanistic views of ecology (such as Leopold's injunction to keep every cog and wheel [104] or Ehrlich & Ehrlich's disappearing aeroplane rivets metaphor [105]), kinship or community among humans and other species (such as in worldviews across cultures [14,16,17,106]), and direct calls for moral consideration of non-human interests (such as arguments for animal rights [107] or criticisms of conservation agendas based purely on human interests [30,108]).

We would expect evolved conservation ethics to promote conservation of keystone species [109], whose structuring effects on ecosystems mean that their *r′* value is likely far more positive than their apparent impacts on human well-being might suggest. This could apply even to species that can be directly harmful (for example, by killing or injuring humans) and indirectly harmful (for example, by competing for prey species) but indirectly beneficial, such as large carnivores or venomous snakes. Human societies often give special protection to keystone species, for example, through formal legal protections for top predators [110] and informal cultural protections for plants that provide food and habitat for a variety of other species [40,87,92,96]. Psychological dispositions such as assigning awe or charisma, reinforced by ascribing special cultural status [76,111], could help conserve dangerous species that pose direct threats but provide net benefits to humans through their broader ecological effects (*r′* > *r*).

### 4.4. Contemporary global conditions

If human societies were always to evolve more or less independently from each other, nuanced, locally adapted conservation behaviours would emerge through individual and social learning and be passed on to successive generations within groups [40,112]. Occasional environmental shocks could further refine locally adapted conservation behaviours [69] and the ethics that support them [95]. Continuous strong feedbacks between human behaviour, ecological consequences and inclusive fitness could mean that ecological knowledge would become richer, conservation behaviours would become more deeply embedded in local traditions, and conservation ethics would become more refined and precisely adapted as a function of residence time. Traditional ecological knowledge is often the product of enduring associations between people, non-human species and ecosystems [14,87], and comprises rich ecological expertise as well as moral components such as correct ways to relate to locally important species and places [94,113–115].

During the past few hundred years, human societies have become larger and more globally interconnected. As a result, people have encountered unfamiliar species and assemblages [116,117], as well as unfamiliar cultural variants such as religious and economic practices whose adoption might impact local ecological and socio-cultural relationships by altering land use, institutions or livelihood strategies [112,118]. Modern technologies have enabled faster and more intensive environmental exploitation (permitting substantially larger values for *x*), often with colossal negative effects on biodiversity [2,4,5]. During or following periods of rapid or substantial change, we would expect mismatches between the species people act to conserve and those it would be most advantageous to conserve. Cultural adaptation could help calibrate conservation behaviours and ethics to new or changing conditions more quickly than genetic adaptation.

Contemporary global conditions, many of which are associated with anthropogenic climate change [2,70], are evolutionarily novel and could have loosened feedbacks between individual behaviours and ecological consequences (for example, melting polar icecaps due to land use change and increased consumption in lower-latitude cities). Contemporary global conditions have also created situations in which people experience negative consequences of others' environmentally harmful behaviour, even when they live far apart (for example, disproportionate costs of sea-level rise, deforestation and biodiversity loss borne by people in lower-consumption regions). Selection pressures associated with ACR will be weaker when feedbacks are looser, because harmful consequences will not necessarily be borne by the people who cause them or their relatives. In extreme cases, when feedbacks are effectively non-existent, ACR may not regulate environmentally destructive behaviour, so selection could favour environmental exploitation and jeopardize the survival of non-human populations and human populations who rely on them.

### 4.5. Collective action for conservation

Our models focus on decisions made by an individual. However, when a focal individual is a member of a group and benefits of conservation are available to other (possibly not genetically related) group members, that individual must incorporate additional considerations into decisions about whether to *do something*. For example, should the focal individual be the first to pay the cost of conservation behaviours? Should the focal individual pay the cost of conservation at all, or let others pay it and still reap the benefits? These are considerations about whether to cooperate with other group members (contribute to the cost of conservation) or defect (not contribute to the cost of conservation) and require game-theoretic analysis. Incentives to defect could delay or derail effective conservation efforts.

Three general collective action scenarios could be particularly helpful in explaining why groups may struggle to conserve non-human populations, species or communities, even when doing so would benefit every individual in the group. In such scenarios, conservation ethics derived from ACR might not be sufficient to motivate conservation. First, prisoner's dilemmas, in which an individual's optimal strategy is to defect unless group members are likely to encounter each other again and are able to keep track of and punish defectors [49,119]. In prisoner's dilemmas, people are more likely to cooperate in small, stable groups and with people whom they are likely to encounter again. However, people are more likely to defect in large, continuously changing groups in which group members rarely or never encounter each other again, making it difficult to track and punish defection [120].

Second, public goods games, in which individuals will cooperate when the factor multiplying the sum of individual investments in an overall public good is sufficiently high relative to group size. In such scenarios, people would be most likely to pay the costs to conserve organisms with higher *r* or *r′* values because those provide largest return benefits. Public goods scenarios are especially relevant because they create opportunities for free-riding, in which defectors reap the benefits of others' cooperation. Efforts to coordinate conservation among diffuse groups of people, for example of organisms with large home ranges or migration routes, would increase opportunities for free-riding. Furthermore, wide-ranging species could have different *r* or *r′* values in different places, and low values in some places would potentially reduce the likelihood of coordinated conservation across large spatial scales. Collective action for conservation has typically been modelled as public goods games or prisoner's dilemmas, revealing how individual defection can generate large-scale environmental degradation [13,119,121–123].

Third, brave leader games [124], in which everyone would benefit from conservation but no one benefits if no one pays the cost. In such scenarios, the initial cooperator pays a disproportionately high share of the cost and risks that cost being wasted if no one else follows. A ‘brave leader’ will eventually emerge when the costs of not cooperating become sufficiently high, but there can be a significant time lag before a brave leader will come forward. After a brave leader does emerge, other cooperators will follow and groups will contain a mixture of cooperators and defectors. Because both lag time and ratio of cooperators to defectors depend on the specific magnitude of costs and benefits, we would expect to see brave leaders emerge sooner and a higher proportion of people within groups willing to conserve organisms with higher *r* or *r′* values.

### 4.6. Implications for value of species debates

Integrating ecology and evolution into our understanding of conservation ethics can help explain why conservation ethics exist, why they vary and why people assign greater value to some species than others. Recent conceptualizations of the value of biodiversity recognize that non-human species have direct and indirect positive effects on human well-being [6,11,60,101]. We formalize such effects in the form of *r* and *r′*. In our models, natural selection operates upon a specific kind of instrumental value that incorporates indirect ecological effects and is measured in inclusive fitness.

Because selectively increasing the success of members of other species can sustain or increase the benefits they provide to humans, altruism towards members of other species may also be altruism towards humans [125]. In a fundamental biological sense, human and non-human interests are not necessarily at odds. This win–win logic could help assuage moral divisions within the conservation community about the extent to which conservation should prioritize benefits to humans or non-humans. An evolutionary perspective suggests that diverse conservation ethics have evolved to promote adaptive cooperation with members of other species in different socio-cultural and ecological contexts. Employing a variety of context-appropriate moral justifications and emphasizing commonalities between conservation ethics [29,33–35] could therefore appeal to a larger and more diverse group of people and so help make conservation efforts more effective. Celebrating and sustaining local traditions, practices and moral justifications for biodiversity [11,115] could ultimately engender more favourable conservation outcomes than promoting universal approaches or single ‘correct’ moral justifications.

## 5. Conclusion

Established scholarship on conservation ethics empirically documents the values people attach to other species and philosophically evaluates moral justifications for conservation. These approaches are important for describing the content of moral beliefs, attitudes, intuitions and norms regarding other species, and scrutinizing assumptions underlying alternative conservation agendas. By integrating ecology and evolution into the study of conservation ethics, we offer complementary insights into the adaptive value of cooperating with members of other species and propose an explanation for the apparent contradiction between widespread conservation ethics and patterns of biodiversity decline globally. A comprehensive evolutionary understanding of why conservation ethics exist, why they vary and their limitations could inform more effective efforts to conserve the diversity of life of which we are part—a moral aspiration across cultures, and a win–win outcome for humans and non-humans.

## Supplementary Material

Appendix A: Measuring model parameters independently

## Data accessibility

This work has no associated data.
