# Supplementary material for: Value of species and the evolution of conservation ethics
Source: R Soc Open Sci. 2018 Nov 21;5(11):181038. doi: 10.1098/rsos.181038 (PMC6281939; doi:10.1098/rsos.181038)
Supplement: Appendix A: Measuring model parameters independently [file rsos181038supp1.docx]

**Electronic supporting material**

**Value of species and the evolution of conservation ethics**

**Darragh Hare, Bernd Blossey, and Kern Reeve**

***Royal Society Open Science***

Appendix A. Measuring model parameters independently

In this manuscript we focus on how our models can explain human conservation behaviours. However, interactions described by our models are not necessarily restricted to humans. They could therefore provide a framework for studying interspecific altruism more generally, as well as the value of one species to another. It would be possible to experimentally manipulate interactions between two species, and measure the value of members of species B (recipient) to members of species A (focal individual). This approach would permit researchers to evaluate, for example, the value of particular plant species to leaf-cutter ants (*Atta* or *Acromyrmex* spp.), the value of particular pollinators to plants they pollinate, or the value of particular prey species to predators.

Researchers could independently measure key parameters from models 1 and 2:

| **What to measure** | **Corresponding model parameter** |
| --- | --- |
| (i) Baseline inclusive fitness of a focal individual who does not invest in recipient and receives no benefit from a recipient | $z$ |
| (ii) Effect of a focal individual’s investment on a recipient’s success (difference between how well the recipient does with or without an investment) | $x$ |
| (iii) Inclusive fitness change (over and above $z$) for a focal individual who receives a return benefit from a recipient but does not itself invest in the recipient | $rx$ |
| (iv) The value in (iii) above, divided by the value in (ii) above ($rx \div x$) | $r$ |
| (v) Difference between the inclusive fitness change of an individual that receives a return benefit but does not invest in a recipient, and the inclusive fitness change of an individual that receives a return benefit and does invest in a recipient | $c$ |
